# Engaging young people within a collaborative knowledge mobilization network: Development and evaluation

**DOI:** 10.1111/hex.13409

**Published:** 2021-12-24

**Authors:** Tanya Halsall, Emma McCann, Julia Armstrong

**Affiliations:** ^1^ Youth Research Unit University of Ottawa Institute of Mental Health Research at The Royal Ottawa Ontario Canada; ^2^ Department of Neuroscience Carleton University Ottawa Ontario Canada; ^3^ Lawrence S. Bloomberg, Faculty of Nursing University of Toronto Toronto Ontario Canada; ^4^ Mental Health and Substance Use Mental Health Commission of Canada Ottawa Ontario Canada

**Keywords:** bioecological model, integrated youth services, mental health, participatory evaluation, youth advisories

## Abstract

**Background:**

It is critical that mental health systems place a focus on prevention and early intervention focused on young people while integrating youth voice to guide priority directions.

**Objective:**

This study was designed to better understand how youth advisories can be utilized to influence strategic directions within integrated knowledge mobilization networks operating within the youth mental health system.

**Design:**

To support this objective, we reviewed the detailed stages of development in establishing a youth advisory within a national network designed to support the integration of youth services. We also engaged the advisory in a participatory evaluation process that examined the extent to which the network had created processes to include youth voice in decision‐making.

**Results:**

Results from the surveys identified moderate to high levels of individual engagement as well as strong development of processes and procedures that support the inclusion of youth voice across the network.

**Discussion:**

Major successes and challenges are presented and discussed with respect to the development of the advisory. The findings are useful for youth advocates and adult allies working to support youth engagement (YE) in knowledge mobilization to enhance the mental health services system. This study also contributes to research and evaluation efforts examining YE and represents an exemplar methodology for evaluating YE efforts at the system level.

**Patient or Public Contribution:**

Young people as mental health service users and youth mental health advocates were involved in the design, data collection, analysis and interpretation of the data as well as the preparation of this manuscript.

## 1 INTRODUCTION

Approximately one in five children and young people experience mental health issues,^1^, ^2^, ^3^ and the majority of lifetime mental illnesses begin before an individual reaches adulthood.^4^ Effective intervention in the early stages of mental illnesses has been associated with better short‐ and long‐term outcomes.^5^, ^6^, ^7^ Recognizing these issues, it is imperative that mental health systems place a focus on prevention and early intervention targeted at youth.^5^ Despite the advantages of reinforcing services for young people, the youth mental health system has been characterized as fragmented and difficult to navigate.^8^ In addition, youth who are entering adulthood typically must transition to adult services, which often results in disengagement and diminished mental health outcomes.^9^, ^10^

Leaders in the field of mental health, including youth advocates, academics and policy‐makers, have identified that services must be transformed to place a focus on individual needs and strengths.^11^, ^12^, ^13^, ^14^ They recommend that this be achieved through collaborative efforts that can provide seamless support throughout the lifespan that incorporate considerations related to a range of developmental influences and contexts. Youth engagement (YE) is one strategy that has been successfully applied to improve outcomes for young people with mental health issues.^15^, ^16^, ^17^ YE is a process whereby young people partner with adults and share their perspective to enhance youth‐focused programmes and policies.^18^, ^19^ Within mental health, YE has been applied to support client empowerment, the design of services, the strengthening of relationships between young people and staff and as a strategy within peer support services.^20^ Models of YE that have been applied within the mental health system include youth advisories^21^, ^22^ as well as involving young people in decision‐making roles on boards.^17^

This study was part of a larger study designed to better understand how YE can be utilized to influence strategic directions within integrated knowledge mobilization created to support the integration of youth services. This paper describes the establishment of a youth advisory and a participatory evaluation process that was designed to examine the processes that helped facilitate the involvement of youth perspective within strategic planning and implementation of an international network. This paper also applies the bioecological model (BEM) to better understand the interactions between the youth advisory and the network and to explain how they might function to influence youth mental health promotion across Canada and the world.

### 1.1 Youth engagement in mental health

YE is a process that integrates the perspective of young people to enhance programmes and policies that are focused on them.^18^, ^19^ This study involves the development of partnerships between adults and young people with the intention of contributing to social change.^23^ YE strategies evolved from an increased focus on the rights of young people as a result of the United Nations Convention on the Rights of the Child.^24^ The convention was created in 1989 and has since become the most highly ratified treaty in history.^25^, ^26^

YE in child and youth mental health has been defined as ‘empowering all young people as valuable partners in addressing and making decisions that affect them personally or that they believe to be important’^27^ (p. 5). Within mental health, YE has been applied to support client empowerment, enhance service design, strengthen relationships among staff and young people and as a strategy within peer support services.^20^ In a review of YE within mental health and substance use services,^15^ researchers identified a range of approaches that can be applied to enhance youth‐focused interventions, including the involvement of young people in programme development.

It is important to note that despite the potential benefits that can be offered through YE, there continues to be many initiatives that fall into tokenistic practices.^28^, ^29^, ^30^ Hart^31^ describes tokenism as ‘instances in which children are apparently given a voice, but in fact have little or no choice about the subject or the style of communicating it, and little or no opportunity to formulate their own opinions’ (p. 9). There are a range of models that have been created to support the development of meaningful YE processes including Hart's^31^ ladder of children's participation, the McCain Model of YE^30^ and the Ontario Centre of Excellence for Child and Youth Mental Health quality standards for YE.^27^, ^32^

Integrated youth service (IYS) models combine mental health and substance use services with community supports, including primary care, housing, vocational and other services.^11^, ^20^, ^33^, ^34^, ^35^ These models are designed to provide seamless holistic supports that can be tailored to client needs and goals. YE is widely practised within IYS models.^36^ In a recent review of youth‐friendly mental health and substance use services,^37^ researchers identified that service integration was a major factor that enhanced the youth‐friendliness of services. In addition, they found that involvement of youth voice was of key importance to inform overall policy and operations, environmental characteristics, staff qualities and service features. This included the formation of youth advisories to guide planning and implementation.

### 1.2 Youth advisories within system‐level initiatives in mental health

There are several examples of youth advisories within Canadian practice. The ACCESS Open Minds IYS research network youth advisory supported site efforts to engage youth within implementation teams as well as to include young people and families on hiring committees.^34^ These efforts resulted in the incorporation of Facebook and text communication, mobile services, providing services in flexible locations and the integration of mental health services alongside other types of services.^38^

Knowledge mobilization networks have adopted youth advisories to inform their strategic direction. For example, Wisdom2Action is a network designed to promote mental health and well‐being for children and young people across Canada.^22^ Wisdom2Action conducted a participatory evaluation that involved interviews and document review to explore the process and impact of YE within the network. They identified that mentorship, clear communication, opportunities for skill‐building and financial and reputational resources were necessary to facilitate effective YE processes. They also identified other themes including the benefits of mentorship, difficulties with retention and the need for flexibility, career development opportunities, investment of resources and to reflect diversity across organizational levels.

### 1.3 The BEM

It is useful to apply the BEM to better understand the functioning of a youth advisory focused on mental health at the system level as it places a focus on the individual's agency over his or her surroundings while also highlighting the reciprocal influence of context on individual development.^39^ The model is based on four major components: (1) process, (2) person, (3) context and (4) time.^39^, ^40^, ^41^ *Process*, or proximal process, symbolizes the increasingly complex reciprocal influence between an individual and his or her developmental context. The *person* component represents both agency and outcomes related to the developing individual. The concept of *time* captures the dynamic nature of continuous development as well as the historical environment that surrounds development. Finally, *context* represents the multiple systems that influence development.^39^, ^42^

Recognizing that the network is functioning at a complex system level, wherein there are multiple levels of influence and dynamic contextual interactions, the BEM provides a conceptual framework through which to operationalize multiple mechanisms and processes, such as examining how advisory members and the network more broadly can influence system‐level outcomes.

### 1.4 Purpose

There is a need for more research that examines youth advisories^43^ and, in particular, how young people can influence organisations functioning at the system level.^44^, ^45^, ^46^ There are several other areas of YE research that are lacking, including studies examining how YE relates to implementation,^44^, ^47^ youth diversity^48^, ^49^ and the development of youth social capital.^48^, ^50^ This study was designed to address the following research questions: (1) how can a youth advisory body be developed to inform organisations working at the system level? (2) How can youth perspective be integrated within strategic directions to inform knowledge mobilization in mental health? We present a detailed description of how a youth advisory was formed within an international knowledge mobilization network. Through a participatory evaluation, we also describe the processes that were developed to integrate youth perspective within strategic planning and implementation of the network to measure the quality of YE. This study applies the BEM^39^ to support the interpretation of the findings to better understand how YE functions to influence youth mental health promotion.

## 2 CONTEXT

Meeting minutes, communications and other operational documents (e.g., YE policy and honoraria process, Terms of Reference [ToR], project planning documents) were reviewed to capture the process of development and significant events. In addition, all three authors were involved in advisory‐related meetings and projects, and McCann was one of the founding Youth Advisory Leads. From this vantage point, they convey the challenges and lessons learned experienced through the multiple stages of development.

### 2.1 Development of representation within the advisory

This study took place in the context of an international knowledge mobilization network that was established to support the uptake and scaling of IYS. The network was established when Canadian leaders in the field of mental health with expertize in the implementation of IYS partnered to leverage their existing work and to identify new ways to increase the implementation of IYS. Early in development, the collaborative leadership, including the founding Youth Advisory Leads, identified that a youth and family advisory should be formed to guide the network. The Youth Advisory was conceptualized as a group of young people working together to provide support to Frayme leadership by drawing on their lived experience to strategically advise and provide input on items of relevance. In addition, their roles involved acting as ambassadors for the network throughout their membership term.

Advisory recruitment was driven by an initial YE committee composed of the two founding Youth Advisory Leads (McCann), one member of the network's Leadership Team and one staff member (Halsall). The committee followed a strategy whereby the candidates were recruited through existing youth advisories from partnering organisations and networks. These organisations all have established youth advisories and are leading the uptake of YE within the Canadian mental health and substance use service system.

Recruitment criteria were developed by the founding Youth Advisory Leads and included the requirement to have previous experience with advocacy and personal lived experience of mental health and/or substance use challenges. In addition, the criteria also included the objective to recruit a set of individuals who reflect diversity based on the following factors: (1) geographic location, (2) ethnicity, (3) age, (4) gender, (5) LGBTQ identities, (6) socioeconomic status and (7) Indigenous identity. Recruits were contacted through their respective organisations, and a summary of the overall network strategy and the general purpose of the advisory was shared to determine interest. The Youth Advisory Leads conducted brief phone interviews with interested candidates to provide more context and confirm interest. Through this recruitment process and a later targeted call, the advisory was formed by 13 members with representation from seven provinces and territories from across the country, coast to coast to coast. Member diversity was also reflected across genders, sexual orientation and cultural groups, including Inuit, First Nations, Métis and newcomers.

### 2.2 Bringing youth voice to the system level

In addition to chairing the advisory, the Youth Advisory Leads worked closely with dedicated staff (Armstrong & Halsall) and participated as members of a decision‐making body created to provide feedback and approval on project development within the network.

From the beginning, the Youth Advisory Leads provided key insight that shaped the development of the network, including organizational structure, strategic focus and policy development. For example, it was one of the Youth Advisory Leads who recommended creating space for two youth positions on the board. This recommendation was critical to prevent youth board members from feeling isolated and to ensure support from each other in situations where they were still learning about key issues. A summary of key Youth Advisory Lead contributions is provided in Table 1.

**Table 1:** Key Youth Advisory Lead contributions

Key Youth Advisory Lead contributions
Providing the recommendation to develop a youth and family advisory to the network leadership
Planning and facilitating the recruitment of advisory members
Codesign of the terms of reference, honorarium process and youth engagement policy
Recommendation for two youth board members aligned with youth advisory
Advocating for the need to engage youth with lived experience of mental health issues
Advocating to maintain separation between the youth and family advisories
Facilitation of all youth advisory meetings
Participation on project planning and approval committees
Coresearchers in participatory evaluation
Coauthor on peer‐reviewed publication

One of the first tasks that youth advisory members engaged with at the outset was the development of the ToR. The draft ToR was developed based on two existing prototypes and then shared with all advisory members, and it was discussed during the first meeting to collect feedback with respect to important details. The advisory members agreed that lived experience should be a qualifying characteristic held by all members. Lived experience did not have to include the experience of receiving services. Advisory members also felt that it was important to build in an option for a leave of absence to accommodate members who might be struggling with their mental health.

#### 2.2.1 Development of the honorarium process

In partnership with the family advisory, an honorarium process was also developed to establish a standard procedure for equitable reimbursement of advisory member contributions (available in Supporting Information Materials). The honorarium process was revised over several months and finally included a tiered format whereby compensation was related to the amount and skill level of work being conducted. Processes were also created to facilitate participation in events through pre‐payment of travel and accommodation costs. Many organisations reimburse travel costs after travel is complete and costs have been incurred. However, these costs can be prohibitive and limit access for many young people who do not have the available funds or a credit card to purchase flights or make a hotel booking.

Similar to the ToR, a prototype YE policy was used to draft the initial policy. The policy was designed to inform the roles and responsibilities of network staff and the Leadership Team with respect to supporting meaningful YE. It elaborated on a range of youth roles, procedures for engaging youth, training, budget requirements, compensation and the creation of positive, safe and accessible spaces.

In the first year of advisory formation, the Youth Advisory Leads conducted strategic planning interviews with individual members to identify their personal objectives for working within the advisory, their ideas for project development and their career aspirations and alignment with advisory work. During these interviews, members also reviewed their current skill sets and examined which skills they were hoping to develop. These included general professional skills such as project management and organisation, written and oral communication, meeting facilitation, negotiation and conflict management.

Initially, it was proposed that one youth member be drawn from the youth advisory to participate on the board, while the other youth board member would be independent. At the outset, this was the case; however, after deliberation, concerns emerged that this strategy might result in misalignment among the youth board members and as a result, the independent youth board member joined the youth advisory. Both youth board members received a brief orientation facilitated by Halsall before their first board meeting and participated in a debrief call after the meeting to discuss their experience and collect their feedback on the process. In addition, before the youth board members' initial meeting, adult board members received a brief presentation about the rationale and importance of YE as well as an orientation to the critical role of youth‐led projects within the network. In addition, an exception was made in the By‐Laws to allow youth and family board members to receive honoraria as acknowledgement for their time contribution.

In its first 2 years, the advisory was involved in a range of projects and activities. Table 2 provides an overview of advisory projects and activities as well as how advisory members were involved.

**Table 2:** Overview of advisory projects and activities

Project/activity	Youth advisory role (e.g., lead, partner, advisor, participant, etc.)
Inaugural convening; initial youth advisory meeting	Participant, advisory leads facilitate
Hiring committee (Director of Operations and Project Coordinator/Youth and Family Liaison)	Participant
Branding committee	Participant
International consultation	advisory leads facilitate
Youth advocacy tool	Lead
Resource on engaging youth and families in network projects	Partner (advising on draft content)
International Initiative for Mental Health Leadership youth match	Partner—network is coleading
Network committee	Participant
advisory evaluation	Colead, participant
Recruitment working group	Lead
Youth engagement policy working group	Lead
Feature profile in the network's newsletter	Participant
Youth peer support request for proposal	Partner/advising on draft content
Stepped care project	Advisor
Quality standards for engagement	Participant
Opioid project	Advisor, participant
IYS scan	Partner supporting data collection + analysis

### 2.3 Challenges

One of the major challenges encountered during the early stages of the youth advisory development was ensuring equitable focus on youth and family engagement. Models of youth and family engagement in youth mental health vary, with some approaches integrating the two perspectives and others maintaining separation between them. It was very important to the Youth Advisory Leads to maintain the youth advisory's independence from the family advisory, and this resulted in some tensions between the two advisories in the early stages of development.

Another challenge that arose was the question of how to ensure that youth and family perspectives were integrated in all network projects and processes in a holistic way. At the outset, several structures and processes existed to ensure that youth and family voices informed strategic planning, through board decision‐making and youth and family involvement on decision‐making committees. Yet, as time progressed, many internal and external projects were initiated without youth and family involvement, and in some cases, youth and family representatives were unable to provide meaningful feedback to refine the project as they were only engaged at a late stage. This process was refined by strengthening connections between the Leadership Team and the advisories by assigning one Leadership Team member to liaise with the advisories. In addition, a formalized review and ranking system was created that allowed projects to be assessed based on meaningful youth and family engagement in a more formal manner.

Finally, recognizing that not all partners were familiar with youth–adult partnerships, challenges emerged surrounding the capacity of partners to create safer spaces that were welcoming, supportive and respectful for young people. Although the network leadership and dedicated staff were highly experienced with and strong advocates for YE, many partners were new to these processes and did not have experience working in collaboration with young people. In addition, advisory members were very intentionally recruited to reflect the diversity of youth voices that the advisory was designed to represent; yet other major partners were involved strategically based on research, policy or practice expertize. As such, during larger events, the diverse representation within the youth advisory stood in contrast with the general lack of diversity across other network stakeholders.

## 3 PARTICIPATORY EVALUATION

### 3.1 Procedure

Early meetings with Youth Advisory Leads led to the decision to follow a participatory evaluation of the network YE strategy. After the recruitment of advisory members, early decision‐making with respect to measures and evaluation questions was facilitated. Advisory members were introduced to the existing research proposal as well as the fundamentals of evaluation. This was followed by a discussion regarding the multiple ways in which members could engage in this study, including being involved only in major decision‐making, actively engaging in research tasks and/or participating as an author on papers of interest.

At the outset, Halsall conducted a review of the literature examining YE research to identify the measures and approaches used to examine YE processes within organisations. A range of instruments were identified including instruments designed to measure YE,^51^ youth–adult partnership,^52^ sociopolitical control,^53^ social responsibility^54^ and youth voice^55^ among others. These tools were presented to advisory members within a spreadsheet that compared validity and reliability scores, methods of collection, variables being measured as well as whether they reflected process or outcome indicators. Advisory members then voted on the tools that they preferred. The tools chosen by the advisory members were the Snapshot Survey of Engagement tool^51^ (SSE) and the Youth Voice at the Agency Level tool^55^ (YVAL). Halsall also facilitated a discussion with advisory members of current issues in youth mental health advocacy. This was used to develop an exploratory design that included new research questions of interest and a photo‐elicitation method.

#### 3.1.1 Survey collection

After selection of YE measures, survey items from the SSE and the YVAL were entered into Survey Monkey and links were emailed to all advisory members in the fall of 2018. Only items from the Head, Heart and Spirit sections of the SSE engagement portrait were included as the other items of the SSE related to descriptions about the activity itself and were not relevant to YE per se. The validity of the SSE has been verified in that psychological engagement was associated with positive experiences within activities and perceived impact related to youth activity involvement.^51^ Previous confirmatory factor analysis has identified strong internal reliability of the YVAL.^55^ Descriptive analyses of the YVAL and SSE are provided.

## 4 FINDINGS FROM THE PARTICIPATORY EVALUATION

### 4.1 Survey results

A total of 12 of the 13 advisory members completed the survey. The average age of the members was 26.4 years, and they ranged in age from 22 to 29 at the time of data collection. One advisory member declined to participate and another did not click the consent box on the survey, so their data were excluded from the results.

In terms of survey results, the average overall score for the SSE was 4.07 out of a possible 5 (Likert responses range from 1—*not at all* to 5—*a lot*). Item scores ranged from 3.3 for *I lose track of time when I'm doing network‐related work* and 4.5 for *I really focus on network‐related work when I'm doing it*. For the YVAL, the average overall score was 3.5 out of 5 (Likert responses range from 1—*least developed* to 5—*fully developed*. YVAL theme scores ranged from 4.4 on *Commitment to Facilitation and Support of Youth/Young Adult (Y/YA) Participation* to 2.4 on *Workforce Development and Readiness to Ensure Meaningful Participation*. Survey scores are provided in Tables 3 and 4.

**Table 3:** Snapshot survey—engagement portrait scores

Snapshot survey item	Average score
I really focus on network‐related work when I'm doing it	4.5
I enjoy doing network‐related work	4.5
Network‐related work connects me to other people	4.4
Network‐related work helps me connect to something greater than myself	4.3
I learn new things when I am doing network‐related work	4.1
I help other people when I do network‐related work	4.1
It would be very hard for me to give up network‐related work	3.9
Network‐related work is an important part of who I am	3.8
Network‐related work helps give my life meaning	3.7
I lose track of time when I'm doing network‐related work	3.3

**Table 4:** Youth Voice Agency Level Assessment (YVAL) theme scores

YVAL theme	Average score
Commitment to facilitation and support of Y/YA participation	4.4
Empowered representatives	3.9
Participation in the evaluation and ensuring programme quality	3.8
Overall vision and commitment	3.7
Collaborative approach	3.4
Participation in developing programming/programme policies	3.1
Leading initiatives and projects	3.0
Workforce development and readiness to ensure meaningful participation	2.4

In addition, the advisory members were asked two open‐ended questions: (1) What is going best in supporting meaningful Y/YA participation in the network? (2) What is most challenging in supporting meaningful Y/YA participation in the network? Advisory members' feedback is provided in Table 5. Paradoxically, communication was highlighted in the process of YE both as a strength and as a major challenge. In addition, guidance from both the Leadership Team and Youth Advisory Leads was noted as being significant assets. Finally, the complexity of the network context and functioning was also recognized as a challenge by several respondents.

**Table 5:** Open‐ended responses to the YVAL survey

Responses to the question: What is going best in supporting meaningful youth/young adult (Y/YA) participation in the network?	Responses to the question: What is most challenging in supporting meaningful Y/YA participation in the network?
Leadership commitment to listening and being responsive to youth perspectives and expertize at all levels	Continuity in the engagement of young people despite geographic barriers and varying participatory styles
Continuing to develop the advisory and the members' roles in the network	Creating official governance structures through policies on how the advisory interacts with the network's board, staff, leadership team and programmes
Staff communication and dialogue	Building rapport and team‐building
Leaders are supportive, understanding and very engaging	Not enough engagements/meetings
The collaboration with the coordinator has been the highlight for myself	For myself I feel information is unclear…
Open communication	having concrete roles for the advisory members
Trying things we ask for and adapting depending on how it goes	feeling distance from the project
Opportunities for the advisory members to contribute to work being done by the network that lies outside of the advisory's direct scope of work	Being kept in the loop about what is going on on a consistent basis
Support and communication from (the advisory leads)	Involvement of Y/YA as equal partners
The leadership are truly invested in the youth perspective and the young people involved in this project.	It's a big project with lots of moving parts, and sometimes it's hard to keep track of them all—makes it hard to feel confident in our participation sometimes
The advisory network is well established and representative. The discussions are open and transparent	Our precise roles or responsibilities are unclear. We are considered, at the broadest level, to strategically advise. However, our meetings seem to consist of having established initiatives that might be approved or disapproved by youth, rather than empowering youth members to contribute their own perspectives. I'm curious about my direct role as a youth member—I wonder if it might be improved by having the ‘adult’ experts guide us on the scope of what is possible, and leave the youth to decide what to do, rather than having us merely approve or disapprove of a project
Bringing everyone at the table physically	The amount of projects proposed w/o action to do it

Abbreviation: YVAL, Youth Voice Agency Level Assessment.

## 5 DISCUSSION

This study was designed to examine the development of a system‐level youth advisory and how it can inform decision‐making and strategic directions to promote youth mental health. This was achieved through the exploration of how the youth advisory was developed as well as the processes created to facilitate YE within the network. Overall, many processes and structures were developed that helped to standardize advisory functioning as well as interactions across the network. Major strengths of YE processes included the diversity of representation that was achieved as well as the systemic view that embedded youth voice across a range of network components and functions. In addition, advisory members perceived YE and the incorporation of youth voice within the network as relatively high. Major challenges were related to balancing youth and family engagement, identifying ways to integrate meaningful YE consistently across projects and creating safer spaces for young people among partners.

The overall level of engagement across advisory members was positive, with an overall score of 4.07 (out of 5) on the SSE and 3.5 (out of 5) on the YVAL, indicating moderate to high levels of individual engagement as well as strong development of processes and procedures that support the inclusion of youth voice across the network. The network was still in a development phase, so not all of the processes and projects were in place at the time of writing, and many individual advisory members had not yet had significant involvement in leading projects or providing direction. As such, it can be expected that scores would likely increase as network implementation continues. In addition, both tools (SSE and YVAL) are designed to be used within agencies that provide direct services to youth and young adults, whereas the network has a main target audience that includes individuals in research, policy, practice and young people and family members working in mental health advocacy. In addition, most major projects do not directly involve young people as a main audience or service recipient. As such, many of the questions did not relate very well to the work that the network undertakes. Relatedly, at the time of data collection, Youth MOVE National, the developers of the YVAL scale, were in the process of developing the Assessment of Youth/Young Adult Voice on Committees and Councils (Y‐VOC). Although the tool was not yet available at the time of data collection, it would likely have been a better fit for this context and will be reviewed for future projects.

Consistent with the survey results, individual members anecdotally shared their frustration with situations where there was limited advisory involvement and communication. Much of the work of the network and advisory is accomplished virtually; therefore, there is relatively limited direct involvement of advisory members in day‐to‐day operations. This may have contributed to difficulties related to communication. For example, the YVAL score on the *Workforce Development and Readiness to Ensure Meaningful Participation* subscale was low. In part, this can be explained by the fact that some of the processes and structures that were in place to involve youth voice were not widely known across advisory members. For example, one of the items in this subscale asks respondents to identify whether young people are involved in the hiring process. Although one or both of the Youth Advisory Leads were involved in the selection of the Director of Operations and the Project Coordinator responsible for liaising with the advisory, many of the scores indicated that this was not a process that was in place. In effect, the process *was* in place; however, not all members were aware of it.

Despite the existence of some processes and structures that were not broadly recognized, advancements can continue to be made within the network with respect to staff and partner training. One of the Youth Advisory Leads identified possible strategies to move forward in these areas, including the development of a formal training for the network staff and partners. Other strategies that were identified included further exploration of knowledge mobilization efforts in health research that focus on consumer engagement, networking with other youth advisories with similar mandates and leveraging other work that is going on with YE at the system level such as with Wisdom2Action and the Centre of Excellence for YE.^22^, ^56^ This approach would be useful for other organisations working to develop YE processes to inform knowledge mobilization and health system reform.

It is also important to note that tensions were identified with respect to focusing exclusively on direct lived experience in representation, as opposed to lived experience acquired as a family member. These issues are relevant when considering overall strategies for youth and family engagement as well as navigating specific situations where one advisory disagrees with the other. Some advocates suggest that youth and family perspectives should be blended so that efforts are more integrated. Yet, when youth and family advisories are combined, adult perspectives may take precedence over youth perspectives. Having youth and family advisories separate follows a similar rationale for having only young people with lived experience on the advisory. When an advisory intended for young people with lived experience also includes other voices (such as family members, caregivers or young people without lived experience), concerns arise that the direct experience or ‘patient’ voice could be marginalized. Patient‐centred efforts must place patients at the centre and provide space for supporting voices that can enhance care alongside.

Organisations may seek to incorporate collaborative leadership models and strategies^57^ to enhance the participation of young people among other stakeholders. These models support decision‐making through consensus rather than majority rule and new ways of bridging cultural boundaries that can create a shared vision that truly engages youth perceptions and applies them to improve effectiveness.

### 5.1 Theoretical implications

The BEM also serves to highlight the significance and potential influence of developmental contexts outside of formal mental health services. When interpreting the interaction of the advisory with the network through the BEM, the concept of the proximal process and related implications are helpful. In Bronfenbrenner's first proposition, he suggests that:

human development takes place through processes of progressively more complex reciprocal interaction between an active, evolving biopsychological human organism and the persons, objects, and symbols in its immediate external environment. To be effective, the interaction must occur on a fairly regular basis over extended periods of time.^39^ (p.797)

It is important to highlight the influence of the network involvement on individual advisory member development as a proximal process that supports their development over an extended period of time and with evolving impact. Advisory members are learning valuable skills, including sophisticated communication and negotiation with leaders in policy, research and practice. They are involved with many projects and activities that provide them with knowledge about system‐level functioning, and they are developing insights with respect to how the system can be transformed. Through these experiences, advisory members are developing significant social capital through connections with high‐level actors (both young people and adults) across organisations and governments. These individuals are leaders in their fields and have the capacity and resources to create new positive opportunities for advisory members to set them on trajectories that may not have been accessible without being involved in these roles. It may be useful to consider the YVAL as a way of measuring the strength of proximal processes in the direction of the potential capacity for advisory members to influence the network direction. Since the tool is designed to measure the processes and opportunities that enhance youth agency over the network strategic directions and operations, it can be conceptualized as measuring the conduit through which the advisory can create organizational‐ and system‐level impacts.

It should also be noted that the Youth Advisory Leads were functionally different from other advisory members as they played facilitation roles during meetings and teleconferences, they took on more responsibility and had more intimate understanding of the functioning and impacts of the network as well as the related YE processes. Since Youth Advisory Leads are more closely involved in processes, meetings and higher‐level decision‐making, their level of engagement is more frequent and extended than other advisory members. Recognizing the potential benefits of frequency, duration and increasing complexity, the benefits accrued through proximal processes would be more significant for advisory members acting as Youth Advisory Leads and to a lesser extent, board members.

The influence of social determinants on mental health and potential interventions that focus on multiple domains, including neighbourhood, environmental and social/cultural, have been recommended.^58^, ^59^ Policy and practice must increase their focus on developmental contexts that can have a significant positive influence on youth development outside of the health system. In particular, creating healthy microsystems where children and young people spend considerable portions of their time should be a key focus in addition to formalized services. This might include, but is not limited to, school‐based initiatives, extracurricular activities, parental supports, initiatives to build community networks, vocational opportunities, urban planning that supports healthy communities and, as represented in this particular case, youth advisories.

### 5.2 Strengths and limitations

Although this study demonstrates several strengths with respect to a methodological approach and contributions to the field of YE, there are also limitations that should be identified. First, there were difficulties interpreting some of the items as the YVAL and the SSE tools are designed to be used within agencies that serve young people. Since the network did not provide direct services, it was difficult for respondents to answer items that were connected to direct service provision. Relatedly, some survey respondents suggested that it would have been beneficial to have a *Does not apply/I do not know* option. In addition, we would like to note that it was difficult to identify how the paper should be delimited with respect to the period of time that is described. We describe activities from the very outset of the network and advisory formation; however, the network and advisory continued to evolve after the writing of this paper. This meant that new developments and challenges emerged that were not captured within this article. It will be important for the network and advisory to continue to share lessons learned as they explore new territory with respect to YE within system‐level knowledge mobilization initiatives.

## 6 CONCLUSION

Youth advocacy within mental health continues to evolve and generate new understandings with respect to the potential of youth–adult partnership and the related benefits. The findings from this study identify the complexity of youth advocacy within the context of a collaborative setting amongst interdisciplinary and intersectoral partners. These lessons can be applied to other youth‐focused initiatives looking to strengthen research validity and intervention impacts through the integration of youth perspectives.

## CONFLICT OF INTERESTS

The authors declare that there are no conflicts of interest.

## Supporting information

Supporting information.

## Data Availability

Data are available on request from the authors.
