# Supplementary material for: Engaging young people within a collaborative knowledge mobilization network: Development and evaluation
Source: Health Expect. 2021 Dec 24;25(2):617–27. doi: 10.1111/hex.13409 (PMC8957748; doi:10.1111/hex.13409)
Supplement: Supplementary file 1 — Supporting information. [file HEX-25--s001.docx]

**Honorarium policy**

This document outlines the guidelines and procedures [the organization] staff follow to ensure honoraria payments are issued in a timely and equitable way.

[The organization] is committed to engaging youth and families (referred to as stakeholders within this policy) as consultants, advisors, reviewers and facilitators (among other roles) to inform and accomplish its work. In order to recognize and value the time, expertise and skills that these stakeholders contribute, [the organization] offers honoraria as a way of saying ‘thank you’. For some, honoraria may facilitate participation by providing an incentive and/or helping to address some of the practical barriers to engagement (such as costs associated with childcare or the need to decline other opportunities).

**Honorarium rates**

[The organization]’s honorarium rates were determined in consultation with youth, families and relevant organizations with similar policies. These rates vary depending on the type of work and level of engagement undertaken by a stakeholder.

- Consultation activities include occasions where stakeholders provide specialized advice, support or direction to [the organization]. These instances typically require minimal preparation time. This may include participating in activities like focus groups, meetings, advisory groups, interview panels, planning tables, product development meetings and/or reviewing documents. For consultation activities, [the organization] pays honoraria at a rate of $25 per hour (including time spent preparing).
- Facilitation activities include occasions where stakeholders are directly involved in the facilitation or delivery of interactive sessions (such as learning events, workshops, webinars, consultations and conference presentations) or public speaking engagements. For facilitation activities, [the organization] pays honoraria at a rate of $40 per hour to recognize the additional skills, capacity and energy required. Note that any preparation time is paid at the consultation rate outlined above.

These rates are not intended to include the cost of travel or travel time where required (e.g. flights, accommodations, meals, etc.). Should an activity require travel on behalf of [the organization], arrangements will be made to prepay or reimburse these expenses separately, in alignment with [the organization]’s travel policy.

|  | **RATE (based on role)** | |
| --- | --- | --- |
| **TIME COMMITMENT** | **Consultation activities** | **Facilitation activities** |
| 1 hour | $ 25 | $ 40 |
| 2 hours | $ 50 | $ 80 |
| 3 hours | $ 75 | $ 120 |
| 4 hours | $ 100 | $ 160 |
| 5 hours | $ 125 | $ 200 |
| 6 hours | $ 150 | $ 240 |
| 7 hours | $ 175 | $ 280 |
| 8 hours (full day) | $ 200 | $ 320 |

**Guidelines and considerations**

- An individual engaged with [the organization] is eligible for honoraria if: they are invited or pre-approved by [the organization] to undertake specific activities on behalf of the network and another organization has not already committed to covering the individual’s time.
- If another organization has committed to covering the cost of an individual’s time engaging in [the organization] activities but the rate they are offering falls below that offered by [the organization], [the organization] will ‘top up’ the individual’s hourly rate to align with what they would be paid by [the organization] alone.
- [The organization] recognizes that the need to provide a fixed address, banking information or a social insurance number may create barriers for some. The [the organization] team is committed to talking to individuals about any barriers they may face and offering assistance where possible.
- Some stakeholders may decline the honorarium offered or may prefer that it be redirected to their employer or another organization. Some individuals may also identify another way that they would prefer to be recognized (such as volunteer hours, authorship on a publication or a reference for future employment). In such cases, [the organization] staff will work with each individual stakeholder to determine the most meaningful way to recognize their contributions.
- To ensure security, [the organization] will not save social insurance numbers in an electronic format and staff will discourage stakeholders from sharing their social insurance number via email (instead offering to communicate over the phone or in person, where possible). The Department of Finance maintains a master list of social insurance numbers in a secure, locked location. This information is kept on file for seven years, at which point it is shredded.
- Note that income is not reported unless an individual earns over $500 in honoraria from [the organization] in a given calendar year. Regardless, individuals are asked for their SIN up front as it can be challenging to collect later.
- [The organization] is committed to ensuring stakeholders are recognized for their contributions in a fast and timely manner. To enable this, staff will identify honoraria needs as soon as they arise and prioritize processing honoraria payments wherever possible.
- In the spirit of transparency, [the organization] staff will communicate openly with stakeholders regarding the timing of their honorarium. For example, when engaging stakeholders for a long-term project, it may be an individual’s preference to receive payments at regular intervals instead of one lump sum at the end of the project.
- Stakeholders who are engaged in a long-term role with [the organization] (e.g. Youth or Family Advisory members) are responsible for tracking and reporting their own hours (using a template provided by [the organization]) on a monthly basis. Reminders are provided on a regular basis, but it is the responsibility of each individual to make note of their work completed.
- [The organization] pays a minimum of one hour for any in-person activities in order to recognize the additional effort often required to engage in these activities.

**Internal process for requesting an honorarium**

- When an engagement opportunity arises, [the organization] staff will articulate the availability of honoraria to each stakeholder involved, clearly communicating the amount available (based on the expected time required), the anticipated turnaround time and any implications. For example:

“In recognition of your contributions, [the organization] will offer you an honorarium of <amount> based on a rate of <$X/hour>. You will receive the payment via direct deposit. If you’re interested, I just need a few pieces of information from you first, including your full legal name, current address, direct deposit information and social insurance number. I need to collect this information because your honorarium would be considered taxable income, which means you’ll receive a T4A for it which you’ll need to claim on your income taxes (just like you would for a job). From the time we receive this information, it typically takes two to four weeks for you to receive your payment.”

- In order to prevent asking stakeholders to repeat information previously provided, [the organization] staff will first verify with the Department of Finance whether the stakeholder in question is already set up for direct deposit. If not, staff will invite the stakeholder to complete the direct deposit form and will request a void cheque or direct deposit form from the stakeholder’s bank (often available through online banking).
- [the organization] staff will request the honorarium in writing to [the organization]’s administrative support, including a description of the contributions being recognized, the amount requested and the appropriate cost centre. An expense claim is then prepared and submitted to [the organization]’s Director of Operations for approval and then onto the Scientific Director for signature. Once approved, the claim is submitted to the Department of Finance for processing.
- Once the honorarium has been requested, [the organization] staff will inform the stakeholder of the expected payment date. For example:

“Thanks again for your contributions to this work. As of today, the paperwork for your honorarium is on its way to our finance department. You can expect to see the money in your bank account within the next four weeks. If you haven’t received your money by then, please contact me directly.”
